# Characterisation of a hand-held CZT-based gamma camera for ^177^Lu imaging

**DOI:** 10.1186/s40658-020-00313-y

**Published:** 2020-07-08

**Authors:** Daniel Roth, Erik Larsson, Anna Sundlöv, Katarina Sjögreen Gleisner

**Affiliations:** 1 Department of Medical Radiation Physics, Clinical Sciences Lund, Lund University, Lund, Sweden; 2 Department of Radiation Physics, Skåne University Hospital, Lund, Sweden; 3 Department of Oncology and Pathology, Clinical Sciences Lund, Lund University, Lund, Sweden; 4 Department of Oncology, Skåne University Hospital, Lund, Sweden

**Keywords:** CZT, Hand-held gamma camera, Molecular imaging, ^177^Lu

## Abstract

**Background:**

Currently, hand-held gamma cameras are being developed for ^99m^Tc imaging, mainly for sentinel lymph node detection. These cameras offer advantages, such as mobility and ease of access, and may be useful also for other applications such as biokinetic studies in animals or for imaging of small, superficial structures in patients. In this work, the suitability of a CZT-based hand-held camera for ^177^Lu imaging is investigated. The energy response of CZT-based detectors combined with the multiple photon emissions of ^177^Lu poses new challenges compared to ^99m^Tc imaging, and a thorough camera characterisation is thus warranted.

**Methods:**

Three collimators (LEHR, LEHS, and MEGP) and three energy windows (55 keV, 113 keV, and 208 keV) are investigated. Characterised camera properties include the system spatial resolution, energy resolution, sensitivity, image uniformity, septal penetration, and temperature dependence. Characterisations are made starting from NEMA guidelines when applicable, with adjustments made when required. The applicability of the camera is demonstrated by imaging of a superficially located tumour in a patient undergoing [^177^ Lu]Lu-DOTA-TATE therapy.

**Results:**

Overall, the results are encouraging. Compared to a conventional gamma camera, the hand-held camera generally has a higher sensitivity for a given collimator. For source-collimator distances below 3 cm, the spatial resolution FWHM is within 6 mm for the LEHR and MEGP collimators. Before uniformity correction, the central field-of-view integral uniformity shows best results for the 113-keV window, with values obtained between 11 and 14%. The corresponding values after uniformity correction are within 3%. Effects of septal penetration are observed but are manageable with a proper combination of collimator and energy window setting. Septal penetration and collimator scatter not only affect the 208-keV window but also contribute with counts in lower windows due to energy-tailing effects. The patient study revealed non-uniform uptake patterns in a region that appeared uniform in a conventional gamma camera image.

**Conclusions:**

The results show that the hand-held camera can be used for ^177^Lu imaging. A 113-keV energy window combined with LEHR or MEGP collimators provides the best image system characteristics.

## Background

Small gamma cameras are currently being developed for imaging of small, superficial structures, with applications for sentinel lymph node detection, scintimammography, and parathyroid imaging [1–3]. The larger field of view (FOV) of conventional general-purpose Anger cameras has been sacrificed in favour of a compact camera head that can be positioned more freely, close to the tissue of interest. Positioning is often done manually, sometimes with the assistance of a gantry, and the short source-collimator distance offers a better spatial resolution than what is achievable by a conventional gamma camera. These specialised camera systems are typically optimised for ^99m^Tc applications. In order to obtain a compact camera head with improved spatial resolution, the detector technologies often differ from the conventional Anger design [2–12].

Small, hand-held gamma cameras may provide benefits for other applications as well. For biokinetic studies, a mobile, easily accessible equipment allows for more frequent imaging sessions and thus a higher temporal sampling in the resulting data. This can be useful for preclinical imaging of small animals, as well as for patients with superficially located structures of interest.

^177^Lu is a *β*^−^-emitting radionuclide of increasing use in radionuclide therapy, including [ ^177^Lu]Lu-DOTA-TATE for the treatment of neuroendocrine tumours [13] and [ ^177^Lu]Lu-PSMA for prostate cancer [14, 15]. ^177^Lu decays with a half-life of (6.647 d) to stable ^177^Hf. In addition to the *β*^−^ emissions, gamma photons are also emitted with energies (yields) of 112.9 keV (6.20%) and 208.4 keV (10.38%) [16]. These emissions are commonly used for gamma camera imaging and dosimetry for patients receiving radionuclide therapy [17]. For measurement of uptake where attenuation is low, such as superficially located structures in patients or for animal studies [11, 12], the X-ray emissions of 54.6 keV (1.59%) and 55.8 keV (2.78%) are also of interest. In the further text, the three resulting photopeaks will be referred to as the 113-keV, 208-keV, and 55-keV peaks, where the latter results from the combined detection of the two X-ray emissions at approximately 55 keV.

The aim of this work is to characterise a hand-held cadmium zinc telluride (CZT)-based gamma camera and investigate its suitability for ^177^Lu imaging. The camera is originally designed for ^99m^Tc imaging, i.e. a single-photon emitter with a gamma energy of 140.5 keV [11, 16]. The energy response of pixelated CZT semiconductor detectors is generally characterised by photopeaks combined with the so-called low-energy tails, caused by trapping of charge carriers and charge sharing between anode elements. A radionuclide such as ^177^Lu presents particular challenges with its multiple photon emissions, where one is of a higher energy (208 keV) and thus interferes with the lower energy photopeaks and energy windows. The investigated characteristics include typical properties of gamma camera systems, such as uniformity, energy linearity and resolution, spatial resolution, and system sensitivity. In addition, the sensitivity of the camera to the internal temperature is investigated, and the ability to shield against ^177^Lu sources located outside the detector FOV that preferably should not contribute to the measured count rate. The CZT technique is now also available in clinical gamma cameras where imaging of ^177^Lu is being investigated [18]. When relevant, comparisons to the characteristics of a clinical Anger-type camera have thus also been made.

## Materials and methods

### Detector characteristics

A CrystalCam hand-held gamma camera was used (Crystal Photonics GmbH, Berlin, Germany), based on a single OMS40G256 CZT semiconductor detector module (Orbotech Medical Solutions, Israel) containing a 39×39×5 mm^3^ crystal [11]. The anode is pixelated with 16×16 pixels using a pixel pad size of 1.86×1.86 mm^2^ and a pixel pitch of 2.46 mm. Further description and characterisation of this, or a similar detector module was presented in [19–21]. The camera is equipped with collimators designed such that each hole is aligned one-to-one with an anode element, and can also be used with a protective cover (open field) with an air cavity in place of collimating material.

Characterisation was made for three collimators: a low energy high resolution (LEHR), a low energy high sensitivity (LEHS), and a medium energy general purpose (MEGP). Collimator characteristics are summarised in Table 1. The outermost collimator walls are thicker, with approximate thicknesses of 2.5 mm.

**Table 1:** Collimator characteristics according to the manufacturer’s specifications

Name	Hole length	Wall thickness	Hole width	Material	Hole shape
LEHR	22.6	0.23	2.23	Lead	Square
LEHS	11.2	0.42	2.04	Tungsten	Square
MEGP	11.5	0.96	1.50	Lead	Circular

All values are given in millimetre

The camera has the options of low-energy mode, for photon energies of approximately 40 to 250 keV, and high-energy mode, for approximately 40 to 1250 keV. The camera characteristics differ slightly between these modes, with greater variation in the response between pixels in the high-energy mode. The mode of primary interest for ^177^Lu is considered to be the low-energy mode, and the high-energy setting was applied only when studying photopeaks with energies above 250 keV.

The manufacturer’s acquisition software includes two image formats, where one is more suited for research purposes and was used herein. This format includes two list text files, one that states the number of counts registered in each multichannel analyser (MCA) channel for each detector pixel, and a paired file stating the interaction energies in kiloelectron volts, according to the manufacturer’s energy calibration. In addition, information regarding the measurement (date, time, acquisition duration, etc.) is stored in a separate text file. This research format is particularly useful for spectrometric measurements and when several energy windows are used. A software was written in IDL (Interactive Data Language, Harris Geospatial Solutions Inc.) to re-arrange the values in the energy-calibrated file into an array *C*(*x*,*y*,*E*), where *C* denotes the recorded counts as a function of position in the camera FOV and energy, *x* and *y* are the integer pixel numbers (0≤*x*,*y*≤15), and *E* is the discrete energy bins of the manufacturer’s energy calibration (*E*=*k*·*ΔE*, *k*=0,1,2,…,*N*_bins_−1). The energy bin width *ΔE* was 0.1 keV and 0.5 keV for the low-energy mode and high-energy mode, respectively. An image *I*_*W*_(*x*,*y*) was generated for an arbitrary energy window *W*={*E*∣*E*_low_≤*E*≤*E*_high_} according to:

1 $$ I_{W}(x,y) = \sum_{E\in W} C(x,y,E).  $$

A spectrum *S*(*E*) was obtained for all pixels, or a subset of pixels *R*, following:

2 $$ S(E) = \sum_{x,y\in R} C(x,y,E).  $$

Since the response varies between pixels, a uniformity correction of the image *I*_*W*_(*x*,*y*) was required and was implemented as correction matrices *H*_*W*_(*x*,*y*) for each energy window *W* according to:

3 $$ H_{W}(x,y) = \frac{\overline{I_{W,\text{ff}}}}{I_{W,\text{ff}}(x,y)},  $$

where *I*_*W*,ff_(*x*,*y*) was calculated using Eq. 1 for an image acquired for uniform detector irradiation using a flat-field phantom (a fillable 8×8×0.7 cm^3^ cavity), and $\overline {I_{W,\text {ff}}} $ is the mean value of *I*_*W*,ff_(*x*,*y*) calculated over all positions *x*,*y*. Uniformity-correction matrices were calculated separately for each combination of radionuclide and collimator. For an acquired image *I*_*W*_(*x*,*y*), a corresponding uniformity-corrected image *I*_*W*,H_(*x*,*y*) is thus obtained as *I*_*W*,H_(*x*,*y*)=*I*_*W*_(*x*,*y*)·*H*_*W*_(*x*,*y*).

#### Temperature dependence

The sensitivity of the spectral response to the operation temperature was analysed by determining the photopeak positions of ^57^Co (122.1 keV and 136.5 keV), ^241^Am (59.5 keV), ^177^Lu (55 keV, 112.9 keV, and 208.4 keV), and ^111^In (171.3 keV), as functions of the detector temperature. The detector was powered on, and the detector’s internal temperature, as displayed by the manufacturer’s software, was logged. Energy spectra were acquired repeatedly during the warm-up of the detector, until thermal equilibrium with the surrounding environment was reached.

Generally, subsequent measurements were made with stable detector temperatures of approximately 29 to 30 ^∘^C. In addition, the spectra for ^177^Lu were also corrected for small energy drifts, by comparing the measured photopeak positions with the true emission energies and determining an acquisition-specific scale factor and offset for the energy scale.

#### Energy linearity and resolution

The energy response was assessed by acquiring energy spectra of several radionuclides and analysing the prominent photopeaks. Acquisitions were made using the open field cover, and spectra were obtained using Eq. 2 and summing over all detector pixels. The radionuclides used were ^99m^Tc (140.5 keV), ^111^In (171.3 keV), ^131^I (284.3 keV and 364.5 keV), ^177^Lu (112.9 keV and 208.4 keV), ^22^Na (511.0 keV), ^54^Mn (834.8 keV), ^57^Co (122.1 keV and 136.5 keV), ^133^Ba (81.0 keV), ^137^Cs (661.7 keV), and ^241^Am (59.5 keV). For radionuclides with a prominent photopeak below approximately 250 keV, spectra were acquired in both low- and high-energy modes and were otherwise only acquired in the high-energy mode. The relative difference between a measured photopeak position *E*_meas_ and the corresponding emitted photon energy *E*_em_ was calculated as (*E*_meas_−*E*_em_)/*E*_em_.

The energy resolution was determined using two procedures. In the first, the energy resolution full width at half maximum (FWHM) was calculated according to the NEMA NU 1-2012 standard [22], where the energies corresponding to the half peak heights were determined using linear interpolation. In the second procedure, a Gaussian function was fitted to the peak. Because of the charge transport properties of the CZT crystal, with its low-energy tails, the obtained energy spectra were not well described by Gaussian functions. In order to exclude the tail from curve fitting, Vadawale et al. [20] used a Gaussian function defined in an energy interval that only contained the high-energy side of the photopeak. In this work, a similar approach was used, with the modification that part of the low-energy side of the photopeak was also included in the curve fitting. The energy resolution estimation was made in a two-step process. First, a function with the form:

4 $$ S_{1}(E) = a_{0} \cdot e^{-\frac{1}{2}\left(\frac{E-a_{1}}{a_{2}}\right)^{2}} + a_{3} + a_{4}\cdot E + a_{5}\cdot E^{2}  $$

was fitted in an energy interval [*E*_*l*_,*E*_*u*_] over the photopeak, where the interval typically ranged from −3 to +10% of the emission energy. A second energy interval [*a*_1_−*p*·*a*_2_,*E*_*u*_] was then defined, with the lower energy limit adjusted using the parameter *p*. A function with the form:

5 $$ S_{2}(E) = a_{0} \cdot e^{-\frac{1}{2}\left(\frac{E-a_{1}}{a_{2}}\right)^{2}} + a_{3},  $$

was fitted, and the energy resolution was then calculated as the Gaussian FWHM ($ 2\sqrt {2\ln 2}\cdot a_{2} $). The energy resolution was described as a function of the emitted photon energy, following:

6 $$ \text{FWHM}(E) = A + B\cdot E.   $$

Values for *A* and *B* were determined by linear regression. Initial estimates for the function fits were derived by data-driven methods, as described in Appendix 1.

#### Intrinsic count rate performance

The count rate performance was investigated by placing the detector at a 5-cm distance from the flat-field phantom filled with ^99m^Tc. The open field cover was used. The count rate in the whole energy spectrum and in a 140.5-keV ±7% energy window was recorded as a function of the activity, with an initial activity of approximately 8 MBq.

### Characterisation for ^177^Lu

#### Flat-field uniformity

The uniformity in the response across the detector FOV was evaluated using flat-field reference measurements acquired for uniformity-matrix calculations (Eq. 3). These were acquired with the flat-field phantom filled with [ ^177^Lu]Lu-DOTA-TATE and the detector placed at a fixed source-collimator distance of 5 cm. For all measurements, the average count level exceeded 50,000 counts/pixel for all energy windows of interest. The relative deviations from the mean, as reflected by the uniformity-correction matrices *H*_*W*_(*x*,*y*) in Eq. 3, were analysed for each energy window setting and collimator (including the open field cover). As summarising metric, the integral uniformity was calculated following the NEMA standard as (max(*I*_*W*,ff_)− min(*I*_*W*,ff_))/(max(*I*_*W*,ff_)+ min(*I*_*W*,ff_)). The subsampling to a target pixel size (6.4 mm) and low-pass filtering indicated in NEMA [22] were omitted due to the small FOV. Differential uniformity calculation was also omitted for this reason. The integral uniformity was calculated for the central and full FOV (14×14 pixels and 16×16 pixels), as border pixels theoretically have a different response [23]. The integral uniformity was also analysed after uniformity correction, using another set of flat-field measurements. The average count level exceeded 15,000 counts/pixel for these images, yielding a relative standard deviation associated with noise below 1% (see Appendix for details). In addition, the energy spectral response for a selection of pixels, representing different levels of deviation, was analysed.

#### System spatial resolution

The spatial resolution was determined for the three collimators, following the general procedure outlined in NEMA NU 1-2012 for discrete pixel detectors. A thin line source was used, constructed by filling a glass micropipette tube (diameter 1.1 mm, length 10 cm, volume 100 *μ*l) with a solution of ^177^Lu. Measurements were made at source-collimator distances between 0 and 140 mm. For each distance, measurements were made for 10 lateral positions by moving the line source in steps of 1 mm. Raw data were stored, and images generated by applying the appropriate energy window and uniformity correction. Line profiles were drawn in the central FOV, and the FWHM was determined using linear interpolation to find the positions where the profile intersected half the peak height. The mean of the 10 FWHMs was taken to represent the spatial resolution for the given collimator, energy window, and source-collimator distance.

In order to set the results of the hand-held camera in relation to those of a conventional gamma camera, the spatial resolution was also determined for a clinical Tandem Discovery 670 system (GE medical systems). Four line sources filled with [ ^177^Lu]Lu-DOTA-TATE were imaged simultaneously, two aligned along the *x*-axis and the other two along the *y*-axis. Imaging was made using an MEGP collimator and a 15% wide energy window over the 208-keV photopeak. A 512×512 image matrix was used with a pixel size of 1.1 mm. Measurements were made for both detector heads at source-collimator distances between 11 and 84 mm. The spatial resolution was calculated for each line source using the interpolation procedure, and a final spatial resolution for each distance was obtained as an average across all line sources and detector heads. To determine the spatial resolution over a greater span of distances, the physical measurements were complemented by Monte Carlo simulations [24]. The imaging parameters, camera specifications, and collimator specifications were set to emulate the Discovery 670 measurements. One single line source was simulated at a time, aligned with either the *x*- or the *y*-axis, at source-collimator distances between 1 and 150 mm.

#### System sensitivity

In order to investigate the system sensitivity, a resealable phantom was constructed with a diameter smaller than the detector FOV and consisted of a cylindrical cavity (diameter 20 mm, height 8 mm) with 5-mm-thick PMMA walls and a top and bottom thickness of 1 mm. The phantom was filled with [ ^177^Lu]Lu-DOTA-TATE with activities of approximately 60 MBq, measured using a traceable Secondary Standard Dose Calibrator (Southern Scientific, Henfield, UK). The phantom was placed in the centre of the detector FOV, 3 cm from the collimator face, and images were acquired with the MEGP, LEHR, and LEHS collimators. The system sensitivity *ε*_*W*_ for an energy window *W* was calculated according to:

7 $$ \varepsilon_{W} = \frac{c_{W}}{t \cdot A}\cdot \frac{t\cdot \lambda}{1-e^{-t\cdot \lambda}},  $$

where *c*_*W*_ is the number of counts within the full FOV or within a region of interest (ROI) in the image *I*_*W*,H_(*x*,*y*) of the sensitivity phantom, *t* is the elapsed time of the measurement, *A* is the activity in the phantom at the start of the measurement, and *λ* is the decay constant of ^177^Lu. Two approaches were used to determine *c*_*W*_. The first used the full FOV as indicated by NEMA, while the second used a circular ROI for calculation of *c*_*W*_. The ROI was introduced to obtain a measure of the sensitivity that was less dependent on the FOV dimensions. This ROI was generated with a centre point determined as the centre of gravity of the count distribution, and a radius *r*=*r*_source_+0.5·FWHM(*d*), where *r*_source_ is the phantom radius (10 mm) and FWHM(*d*) is the collimator spatial resolution at distance *d*.

According to NEMA NU 1-2012, the magnitude of septal penetration and scatter can be estimated by measurement at various distances. This procedure was found to be less applicable owing to the small size of the detector and source combined with the poor spatial resolution at large distances. Instead, effects of septal penetration and scatter were qualitatively assessed as the sensitivity as a function of the circular ROI radius.

#### Shielding, septal penetration, and collimator scatter

The detector’s ability to shield against ^177^Lu sources located outside the FOV was assessed according to the NEMA standard for the MEGP and LEHR collimators. The detector was positioned facing downwards, and a vial of ^177^Lu was placed at a source-collimator distance of 20 cm. The vial was then moved laterally, and the count rate determined as a function of the lateral distance for each of the ^177^Lu energy windows.

The effect of septal penetration, including collimator scatter, was investigated by acquiring spectra of ^177^Lu in the flat-field phantom, using the MEGP, LEHR, and LEHS collimators and with the open field collimator. A fixed source-collimator distance of 5 cm was used. The relative heights of the 113-keV and 208-keV peaks were compared qualitatively to estimate the amount of septal penetration at 208 keV. Effects of septal penetration and collimator scatter were also assessed using the sensitivity measurements, as described above.

#### Patient measurements

To assess the practical applicability of the hand-held detector in a patient situation, images of a tumour, localised in the patient’s upper arm, were acquired for a patient receiving [ ^177^Lu]Lu-DOTA-TATE therapy. Imaging was made with the MEGP and LEHR collimators (1 day) after administration of 7.4 GBq [ ^177^Lu]Lu-DOTA-TATE. The acquisition time was 2.5 min, which for the 113-keV energy window yielded a total of approximately 12,000 counts and 20,000 counts, for the MEGP and LEHR collimators, respectively. To reduce the photon exposure of both the operator and the detector, a lead shield was placed between the patient’s torso and arm.

For comparison, the gamma camera images acquired on the same day as part of the standard clinical procedure were also analysed. These images consisted of a planar anterior-posterior whole-body scan and a CT scout image, acquired using the Discovery 670 SPECT/CT system. An MEGP collimator was used, and acquisition made in a 15% energy window centred on the 208-keV photopeak. For localisation purposes, the whole-body gamma camera image was co-registered to the CT scout image. For further details on the acquisition protocol, see Sundlöv et al. [25].

## Results

### Intrinsic count rate performance

The count rate was found to be linear with the ^99m^Tc source activity for both the 140-keV energy window and for an energy window spanning the full measurable range (0 to 250 keV), up until a count rate of approximately 71,000 cps in the wide energy window. For higher activity levels, the count rate remained constant. The rate of data transfer between the detector and the host computer (i.e. the USB connection) has been specified as the limiting factor. No count rate losses (e.g. non-linearity with paralysable or non-paralysable behaviour) were observed for count rates up to 71,000 cps.

The maximum amount of ^177^Lu activity that can be adequately measured was estimated using the images acquired from the sensitivity phantom (see the ‘System sensitivity’ section). The count rate was recorded in a wide energy window (0 to 250 keV) and over the whole image, giving sensitivities of 99.8 cps/MBq, 225.9 cps/MBq, and 663.3 cps/MBq for the MEGP, LEHR, and LEHS collimators, respectively. Given these sensitivities, the amount of activity required to reach the count rate limit of 71,000 cps was estimated to be 711 MBq, 314 MBq, and 107 MBq for the MEGP, LEHR, and LEHS collimators, respectively.

### Energy linearity and resolution

The energy response, using the manufacturer’s energy calibration, was found to be linear with the photon energy. The relative deviations in the energies of the eight analysed photopeaks for low-energy mode were (mean ±SD) −0.36±0.57% (−1.05% max deviation), while for the 13 peaks analysed for high-energy mode, the corresponding deviations were −0.16±0.66% (+1.16% max deviation).

**Fig. 1:**
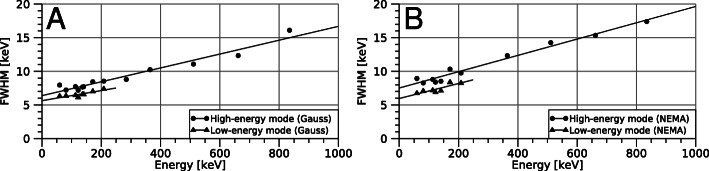
Energy resolution as a function of the photon energy for acquisitions made in low-energy mode and high-energy mode. Results determined by fitting of Gaussian functions (**a**) and by the NEMA interpolation procedure (**b**) are shown. Markers indicate the measured values, while the lines show the linear regressions (Eq. 6)

Figure 1 shows the energy resolution for the low- and high-energy modes determined using Gaussian fits and by the NEMA interpolation procedure. The obtained line parameter values (Eq. 6) are presented in Table 2. The FWHM was slightly larger when determined according to NEMA, as a result of the wider low-energy side of the peaks due to low-energy tails and Compton scattering [20].

**Table 2:** Obtained parameter values for the energy resolution curve (Eq. 6)

	Gaussian fit		NEMA	
	*A* [keV]	*B* [unitless]	*A* [keV]	*B* [unitless]
Low-energy mode	5.64	0.00751	5.96	0.0111
High-energy mode	6.39	0.0103	7.52	0.0121

### System flat-field uniformity

**Fig. 2:**
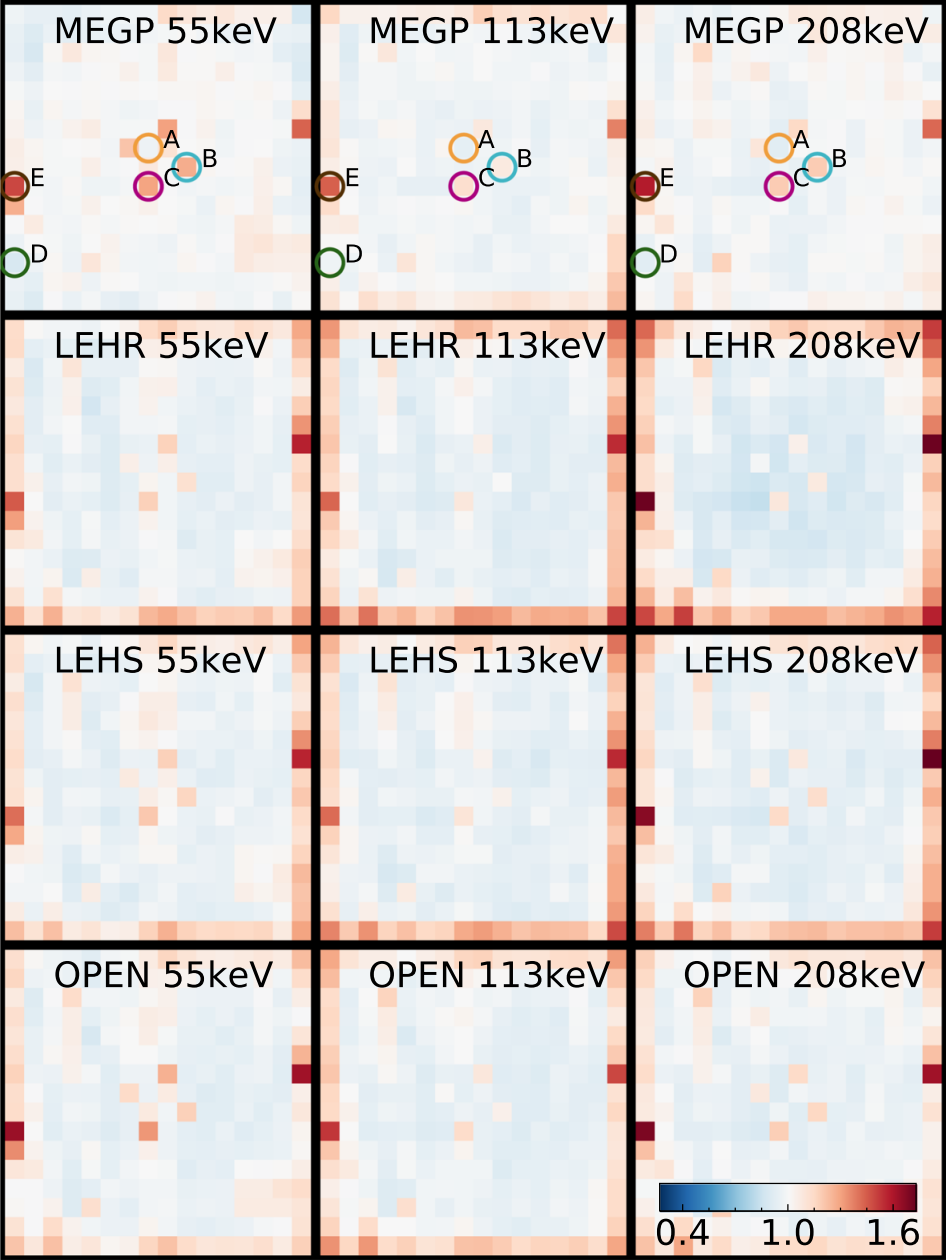
Uniformity-correction matrices for ^177^Lu for each energy window and collimator (including open field). Values higher than 1.0 correspond to anode elements whose sensitivity is below the detector average. All uniformity matrices are shown using the same colour scale (lower right corner). Circles and letters A–E indicate positions of the five pixels selected for spectral analysis in Fig. 3

Figure 2 shows the uniformity-correction maps *H*_*W*_(*x*,*y*) for each collimator and energy window for ^177^Lu, and Table 3 gives the associated integral uniformities before and after uniformity correction. As seen in Fig. 2, the central FOV has a relatively uniform detector response with correction values close to unity for most pixels. However, there were a few centrally located pixels whose response differed, thus giving rather poor quantitative values. The 113-keV window exhibited lowest non-uniformity (Table 3). For the LEHR and LEHS collimators, the response was generally lower at the FOV border, while for the MEGP collimator, this feature was less prominent (Fig. 2) although the lower response of one pixel yielded a higher non-uniformity (Table 3). After uniformity correction, the integral uniformity in the central FOV was within 3% for all collimators and within 4% for the full FOV (Table 3).

**Fig. 3:**
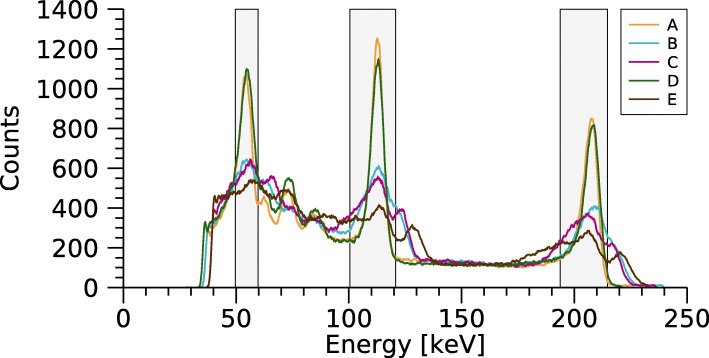
Spectra for the five selected pixels for the MEGP collimator, where letters correspond to positions indicated in Fig. 2. Energy windows are shown as grey rectangles. A 1-keV-wide mean-value filter has been applied for noise reduction

**Table 3:** Integral uniformity (in %) for each collimator and energy window for ^177^Lu, for unprocessed and uniformity-corrected images

		Central FOV (14×14 pixels)			Full FOV (16×16 pixels)		
		55 keV	113 keV	208 keV	55 keV	113 keV	208 keV
	MEGP	19.0	11.1	12.7	26.1	23.2	26.6
Without	LEHR	15.2	11.3	19.5	28.8	27.4	35.2
correction	LEHS	15.2	11.3	14.4	28.0	27.5	32.8
	OPEN*	22.4	13.5	16.5	32.5	28.4	32.3
	MEGP	2.7	2.5	2.6	2.7	3.0	3.3
With	LEHR	2.9	2.5	2.4	3.5	3.8	3.4
correction	LEHS	1.8	1.5	2.5	2.9	2.5	3.1
	OPEN*	1.1	0.9	1.5	1.2	1.0	1.5

*Open field cover

The energy spectra for the five selected pixels are shown in Fig. 3. The pixel selection was made based on the uniformity-correction matrix for the MEGP collimator and 208-keV energy window (Fig. 2), and concerned pixels in the central FOV exhibiting the lowest (A) or highest values (B, C), and pixels on the image border with the lowest (D) and highest values (E). As noted, both the photopeak amplitudes and the energy resolution differed between the pixels, while the amplitudes of the low-energy tails in the 130- to 180-keV range were similar.

### System spatial resolution

**Fig. 4:**
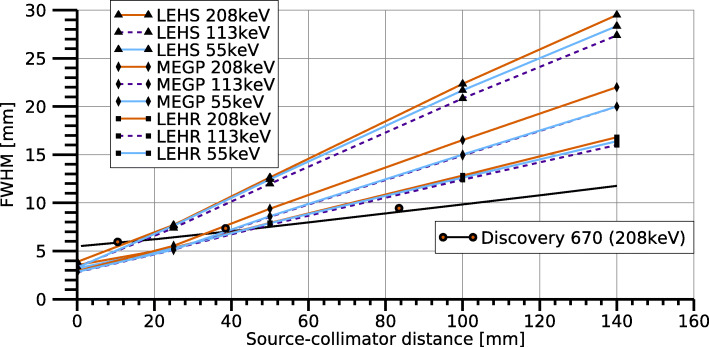
System spatial resolution for ^177^Lu as a function of the source-collimator distance for each collimator and energy window. The spatial resolution for a conventional gamma camera equipped with an MEGP collimator is also shown, with measured (coloured circles) and simulated (black line) data

Figure 4 shows the spatial resolution for ^177^Lu as a function of the source-collimator distance for the MEGP, LEHR, and LEHS collimators. At short distances, the spatial resolution was limited by the dimensions of the anode elements and the collimator hole pitch (2.46 mm). For distances below 3 cm, the resolution was similar for the LEHR and MEGP collimators and all energy windows used, while it was poorer for the LEHS collimator. At larger distances, the LEHR collimator gave the best resolution, especially when combined with the 113-keV energy window. Figure 4 also shows the spatial resolution for a conventional gamma camera (Tandem Discovery 670) equipped with an MEGP collimator. The hand-held gamma camera equipped with an MEGP or LEHR collimator thus yielded a better spatial resolution than the conventional camera at distances shorter than approximately 40 mm.

### Temperature dependence

The internal temperature of the detector was found to increase over a time period of 1 to 2 h after being powered on. The temperature can be described by the function *T*(*t*)=*T*_0_+*ΔT*·(1− exp(−(*t*−*t*_0_)· ln(2)/*t*_rise_)), where *ΔT* is the temperature increase and *t*_rise_ describes the rate at which the detector heats up. The parameter values were obtained to *ΔT*=9^∘^*C* and *t*_rise_=30 min.

**Fig. 5:**
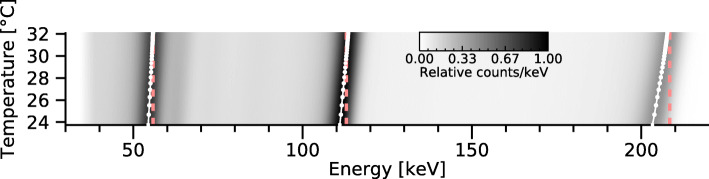
Measured ^177^Lu spectra as a function of the detector temperature. The grayscale intensity represents the spectral amplitude; each row in the image represents one acquired spectrum. White lines shows the photopeak centres, and vertical red lines indicate the reference photon energies

Measured photopeak energies were found to shift towards higher energies as the detector temperature increased during warm-up. A linear relationship between measured photopeak positions and temperature was observed, with peak drifts between 0.17 keV/ ^∘^C for 55 keV and 0.59 keV/ ^∘^C for 208 keV and intermediate drifts for photopeaks between these energies. Figure 5 shows the impact of temperature on measured ^177^Lu spectra, and a table of the drifts obtained for each energy is provided in Appendix.

### System sensitivity

Table 4 shows the system sensitivities for ^177^Lu obtained for the three collimators. For the MEGP collimator, the circular ROI sensitivity for the 208-keV energy window was 74% of that of the 113-keV window, whereas for the LEHR and LEHS collimators, the sensitivities were similar for these energy windows. This suggests that the detector sensitivity at 208 keV for the LEHR and LEHS collimators is elevated due to septal penetration. This effect is even more pronounced for the full FOV sensitivity values.

**Table 4:** System sensitivity (cps/MBq) for each collimator and energy window for ^177^Lu

	Circular ROI			Full FOV		
	55 keV	113 keV	208 keV	55 keV	113 keV	208 keV
MEGP	12.8	17.6	13.0	14.0	19.1	14.7
LEHR	20.0	27.5	26.0	24.2	32.2	43.5
LEHS	61.7	84.4	83.3	72.6	96.7	127.2

**Fig. 6:**
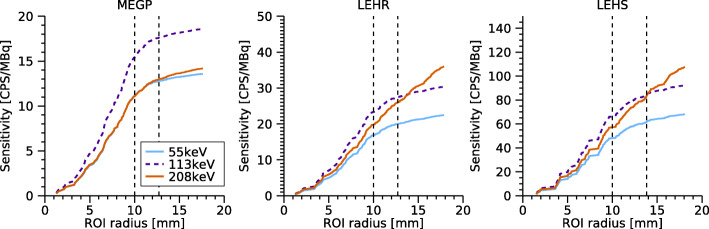
Sensitivities for the three collimators and the three energy windows for ^177^Lu, as a function of the ROI radius. The radii corresponding to the source size, and the source size plus 0.5 times the FWHM are shown as vertical dashed lines

Figure 6 shows the detector sensitivity as a function of the ROI radius used for outlining the sensitivity phantom. For LEHR and LEHS collimators, there were fewer image counts located outside the source for 55-keV and 113-keV peaks, and the sensitivity levelled off for ROI radii greater than the source dimensions. For the 208-keV peak, the sensitivity increased for larger radii, thus indicating effects of septal penetration for these collimators. A slight sensitivity increase beyond the source radius was also seen for the MEGP collimator and the 208-keV peak; however, this effect was less pronounced.

### Shielding, septal penetration, and collimator scatter

**Fig. 7:**
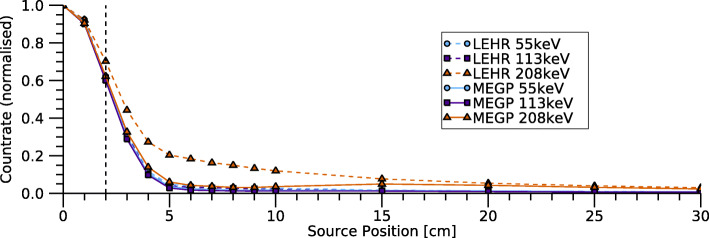
Relative count rate in the FOV as a function of the lateral distance from the detector centre to a ^177^Lu source. The FOV border is indicated by the vertical dashed line

Figure 7 shows the relative count rate from a ^177^Lu source placed at different lateral positions with respect to the detector centre. Since the detector FOV is approximately 4×4 cm^2^, the 2-cm position corresponds to the FOV edge. As a source-collimator distance of 20 cm was used, the limited spatial resolution had a blurring effect on the profiles. When using the LEHR collimator, there was a larger contribution to the 208-keV energy window from source positions outside the FOV, thus indicating that the detector shielding and outermost collimator septa are less suited for discrimination of these photons. For the MEGP collimator and all energy windows, as well as LEHR and 55 keV and 113 keV, the contributions from sources located beyond the FOV edge were minor (less than 6% at distances beyond 5 cm). In practice, the relative contribution from sources located outside the FOV will also depend on the source-to-background activity ratio.

**Fig. 8:**
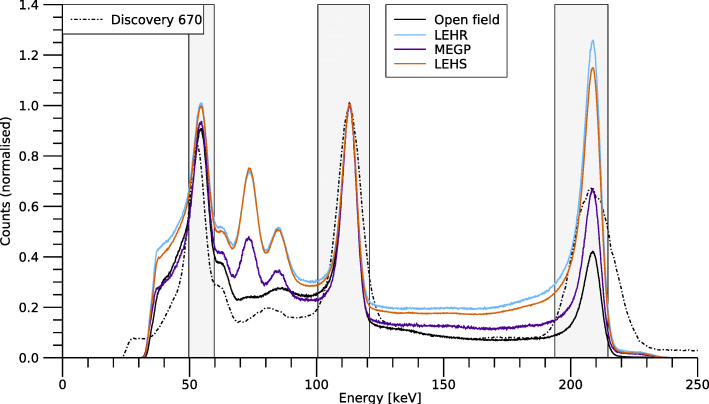
Spectra of ^177^Lu acquired using the open field cover and the three collimators. The spectra are normalised to unity at the 113-keV peak. Differences in peak amplitude may be indicative of septal penetration. A spectrum for the Discovery 670 system is shown for comparison

Figure 8 shows spectra acquired from ^177^Lu in the flat-field phantom with the different collimators and without collimator (open field cover). The figure also shows the three energy windows used for ^177^Lu throughout this work. The spectra were normalised to unity at the 113-keV peak, and differences in the amplitude of the 208-keV peak indicated effects of septal penetration. For comparison, Fig. 8 also shows a spectrum acquired with the Discovery 670 system without a collimator.

### Patient measurements

**Fig. 9:**
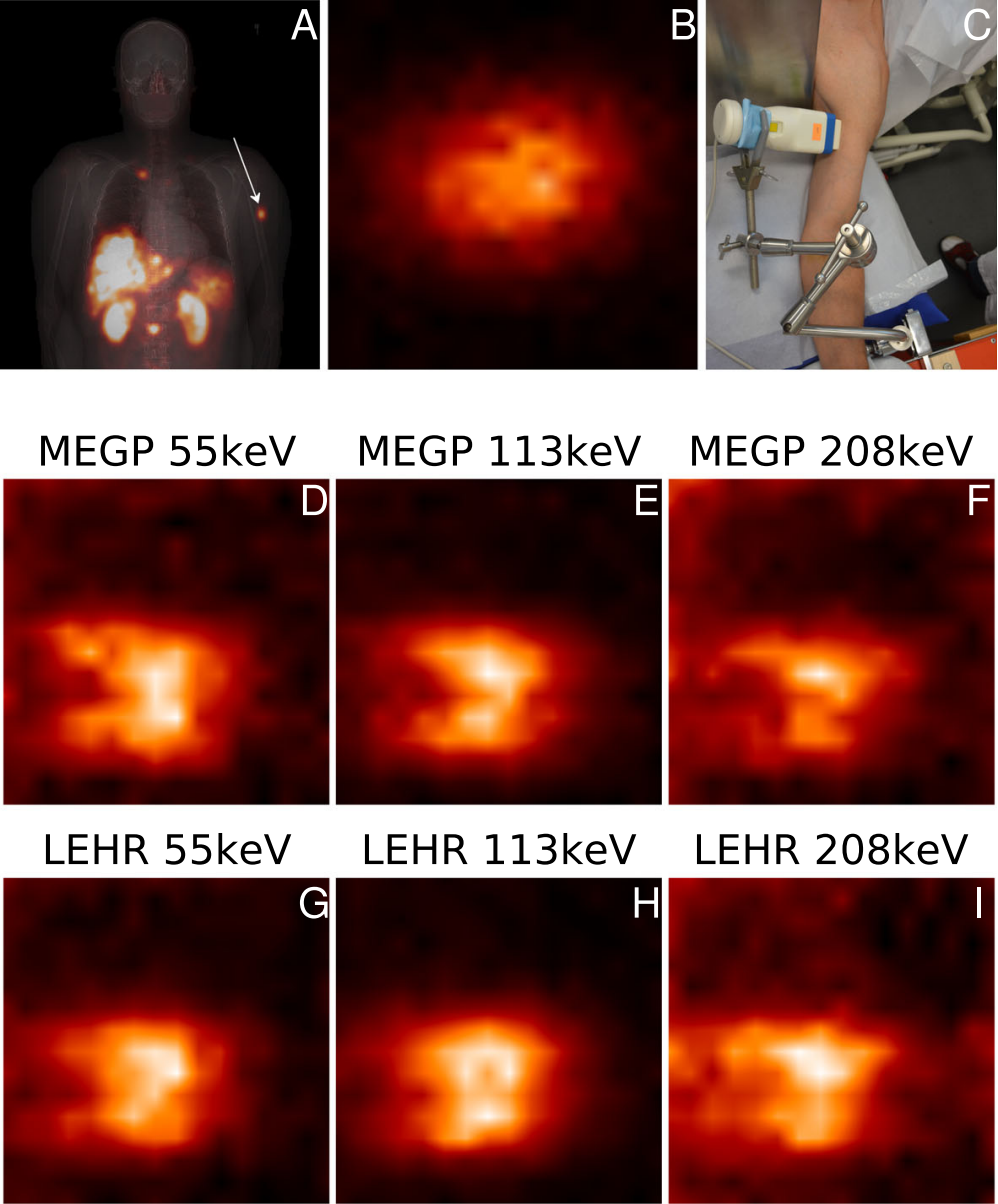
Patient uptake analysed using the conventional and hand-held gamma cameras. Images acquired using the Tandem Discovery 670 system are shown in **a** and **b**. **a** The fusion of a whole-body and CT scout image. An arrow indicates the uptake selected for imaging. **b** A magnification of this uptake in the anterior whole-body projection. The enlarged image spans an 4×4 cm^2^ area and has been rotated such that the distal direction is to the right. **c** A CrystalCam measurement. CrystalCam images acquired with this orientation are shown in **d**–**i**, wherein the distal direction is also to the right

Figure 9 shows the setup for measurement with the hand-held camera. The tumour localisation is also shown in the gamma camera image acquired with the conventional system. In order to make a visual comparison of the images from the two systems, the image from the conventional system has been cropped to an area equal to the size of the FOV of the hand-held system (4×4 cm^2^). However, as shown by the measurement setup, it should be noted that the projection directions differed slightly between the cameras.

Patient images acquired with the CrystalCam system using the MEGP and LEHR collimators and the three energy windows are also shown Fig. 9. Indeed, the tumour uptake of [ ^177^Lu]Lu-DOTA-TATE appeared to be more non-uniform than revealed by the conventional gamma camera image. When comparing the different energy windows, there were slightly more structural details in the tumour region in the 113-keV window. The counts along the image border, especially prominent in the 208-keV window and for the LEHR collimator, result from photons that emanated from outside the FOV and penetrated the detector shielding. Also for the 55-keV energy window, there were counts along the border. The lower yield of 54.6-keV and 55.8-keV photons, combined with a greater attenuation for these lower energies and low-energy tailing, may explain why these counts were more apparent for the 55-keV energy window than for 113 keV. The background count rate was lowest when using the 113-keV energy window, thus giving the highest contrast. Owing to the higher system sensitivity of the LEHR collimator, the total number of counts was approximately 60% (113 keV) to 100% (208 keV) higher than when using the MEGP collimator, and the choice between these two collimators thus mainly depends on the expected count rate.

## Discussion

In this work, a hand-held CZT-based gamma camera is characterised for the potential application of in vivo imaging of ^177^Lu-labelled compounds. Originally, the detector is intended and optimised for ^99m^Tc [11], and a principal question is whether imaging for higher photon energies is feasible and to what extent the image quality is degraded. In this regard, ^177^Lu presents challenges with its two gamma energies at 113 keV and 208 keV, as well as characteristic X-rays at about 55 keV of possible interest for preclinical studies in small animals. Overall, the obtained results show that imaging of ^177^Lu works well, with some image-degrading effects for certain collimator and energy window combinations.

The earlier versions of the NEMA NU 1 standard were intended for scintillation cameras with large crystals. More recent versions also accommodate discrete pixel detectors, but do not explicitly include small-FOV cameras. For the characterisation measurements made herein, the NEMA NU 1-2012 was used as the starting point, but was modified when needed due to the small FOV of the hand-held camera.

A general feature of CZT detectors is the presence of low-energy tails in the energy spectrum. These tails are caused by the relatively poor mobility of electrons and holes in conjunction with charge trapping, which results in a depth dependence for the total induced charge measured by the readout electronics [26]. The subdivision of the anode into smaller elements reduces this depth dependence, but introduces the possibility of charge sharing between anodes [27] which also contributes to the low-energy tail.

For the detector module used, it appears that the anode elements within a given module can be somewhat unique [11, 19, 20] and possess a unique energy calibration. Kotoch et al. [19] and Knoll et al. [11] found that their detectors had a linear relationship between the photopeak channel numbers and the emission energies, while Vadawale et al. [20] described their detector as reasonably linear in the 30- to 150-keV energy range, with some non-linearity for higher energies. In this work, the linear energy calibration (unique for each element) supplied by the manufacturer was used. The observed photopeak positions were compared with the corresponding emission energies, with maximum deviations obtained of −1.05% (low-energy mode) and 1.16% (high-energy mode), thus indicating that the manufacturer’s energy calibration is sufficient.

In addition to the variability across the detector module, the energy calibration depends on the detector temperature. As shown in Fig. 5, the photopeaks drift towards higher energies with increasing detector temperatures, with increasing drifts (in keV/ ^∘^C) for higher photon energies. Since a static energy calibration was used, the drift indicates that the peak position measured in MCA channel numbers is increasing. A temperature-dependent energy calibration was observed by Vadawale et al. [20], who described this effect as a property of the readout electronics. This drift can potentially lead to a blurring of photopeaks if the temperature changes markedly during acquisition. When using the detector for imaging, the drift can to some extent be mitigated by choosing sufficiently wide energy windows. However, the system sensitivity may be affected due to the low-energy tails, whose contribution to the energy windows then varies depending on the detector temperature. Therefore, for best results, the detector temperature should be stable when spectroscopic measurements or quantitative measurements are of interest.

Figure 5 shows that the photopeaks reach their corresponding emission energies at a detector temperature of approximately 32 ^∘^C (between 29 and 33 ^∘^C across all studied radionuclides). This detector temperature is higher than generally reached during our measurements and suggests that the manufacturer’s energy calibration was made at a higher ambient temperature. A drift in gain and offset for the readout electronics over time (month- or year-long time scales) could also be a factor, but other investigations have reported a good stability in these properties [19, 20]. For our measurements, an additional energy correction (see the ‘Temperature dependence’ section) was thus required.

The measured energy resolution in Fig. 1 does not appear to follow a $ \sqrt {E} $ relationship, which would be expected if fluctuations in the number of created or trapped charge carriers were the principal factors limiting the energy resolution [28]. Rather, the resolution appears to have a significant offset, and there is little support for any curvature in the data. The offset can be explained by other noise sources affecting the readout electronics, such as leakage currents [19, 20, 28] whose magnitudes are largely determined by the processing methods and materials used when manufacturing the electrodes on the detector crystal [20, 28]. Our detector can be expected to have a poorer energy resolution than theoretically achievable for CZT due to the use of indium electrodes [19], which yield electrode-semiconductor boundary properties that result in greater leakage currents [20]. Detector leakage currents increase with higher temperatures, giving a poorer energy resolution [19, 20]. A model for the energy resolution of a CZT detector was presented by Chen and Wei [29]. Their model was subsequently applied by Pretorius et al. [30], who found that the energy resolution FWHM was underestimated compared to measurements in the 70- to 160-keV energy interval, and an additional Gaussian term was therefore introduced. For this work, where a wider energy interval has been investigated, neither of these energy resolution models were found to work sufficiently well. A possible explanation is that the detector used by Chen and Wei [29] had a single anode giving a different behaviour than that of pixelated anodes [26] and also had platinum electrodes which can yield lower leakage currents and improved energy resolution due to different electrode-semiconductor boundary properties [20, 28]. These differences in detector systems may be the reason that the energy resolution model by Chen and Wei [29] was found to be less suited for our detector type. Conversely, the energy resolution model and parameters obtained herein (Fig. 1) may not be directly applicable to other detectors, owing to the temperature dependency and inter-module variability in energy resolution [19, 20]. The parameters for the energy resolution curve should therefore be applied with caution.

**Fig. 10:**
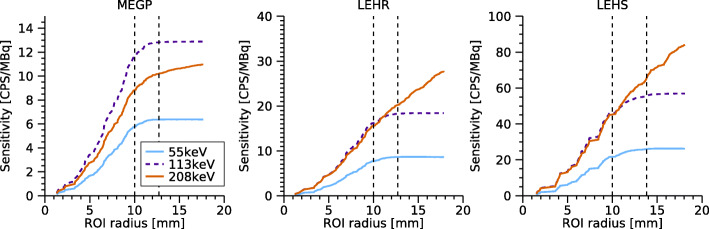
Sensitivities for the three collimators as functions of ROI radius, with TEW correction applied for each energy window. The radii corresponding to the source size, and the source size plus 0.5 times the FWHM are shown as vertical dashed lines

Table 4 shows the system sensitivities obtained for the three collimators. For the LEHR and LEHS collimators, the sensitivity is relatively high for 208 keV compared to 113 keV, indicating effects of septal penetration for 208 keV. This effect is also seen in Fig. 6 where the sensitivity for the 208-keV window increases steadily for radii beyond the source border. Since the detector is optimised for ^99m^Tc and 140 keV, a negligible amount of septal penetration can be expected for the 55-keV and 113-keV photons. However, Fig. 6 shows an increasing sensitivity for radii beyond 14 mm also for these energy windows, which could be interpreted as an effect either of septal penetration or of the low-energy tail of the 208 keV photons. In order to understand the results in Fig. 6, the background counts, caused mainly by the low-energy tail (Fig. 8), were subtracted in a manner similar to scatter correction using the triple-energy window (TEW) method [31], i.e. by placing narrow energy windows on each side of the respective energy window and subtracting a weighted sum of the counts in these windows. Figure 10 shows the resulting sensitivities, which for the 55-keV and 113-keV energy windows become almost constant for radii greater than the source extension. Thus, it can be concluded that the apparent septal penetration for 55 keV and 113 keV in Fig. 6 is mainly caused by septal penetration of 208-keV photons. The results for 55 keV and 113 keV in the shielding measurements (Fig. 7) are affected in a similar manner, and effects of septal penetration for 208-keV photons are also seen in Fig. 8. However, it is noted that charge sharing effects may also play a role, since the low-energy tails have different amplitudes.

With regard to compliance to NEMA NU 1-2012, our sensitivity measurements represent the largest deviation, by the use of an in-house constructed phantom mimicking a small Petri dish, and determining the sensitivity by application of ROIs to the images. The cavity diameter was chosen as a compromise such that the source image would fit inside the FOV with a margin for resolution effects at reasonable source-collimator distances, while simultaneously being large enough to uniformly irradiate several detector elements. Our cavity thickness (8 mm) is greater than what the NEMA standard prescribes (3 mm). The use of non-standard sensitivity sources appears to be common among novel camera characterisations [7–9, 11], with small bottles and point sources as substitutes for a Petri dish. The comparably thick activity layer used for sensitivity measurements (8 mm) is regarded to be a limitation. Count losses due to self-attenuation have been estimated to be approximately 5%, 6% and 8% for 208 keV, 113 keV, and 55 keV, respectively. This means that the detector sensitivity is slightly underestimated, although scattered photons from the phantom may to some extent offset the losses for the lower energy windows. The dimensions of the sensitivity phantom were chosen for practical reasons, to obtain a closed refillable cavity with an activity volume that could be accurately measured in our activity meter. For some collimator and energy window settings, the image counts were non-negligible at the image borders (Fig. 6), meaning that the full-FOV system sensitivity is limited by the FOV dimensions. The ROI was thus introduced to obtain a value of the sensitivity that was less sensitive to FOV size, and is regarded to be a useful complement to the full-FOV sensitivity indicated by NEMA.

The results in Fig. 3 suggest that the main cause of the differences in sensitivity between pixels is variable energy resolution. The presence of a few ‘bad’ pixels with either lower counts or poor spectral properties is expected [19, 20]. Potential causes appear to be defects in the semiconductor crystal and its connections, as well as defects in the readout circuits [32–34]. As seen in Fig. 2, the uniformity-correction matrices for the LEHR and LEHS collimators have higher values on the FOV border and thus a lower sensitivity. A possible cause is septal penetration, which is less pronounced on the border than in the centre due to the thicker outermost collimator walls (approximately 2.5 mm). Another possible explanation is a smaller collection area for anode elements along the image borders [19]. Similar deviations along the image border are present in the uniformity matrix for the open field cover, thus supporting the existence of smaller anode element areas on the module edges. When the MEGP collimator is used, the non-uniformity along the border is less pronounced (Fig. 2). This is probably related to the hole size of this collimator (1.50 mm diameter), which is smaller than the pixel pad size (1.86×1.86 mm^2^) and could thus negate the effects of different anode collection areas. The pixels on the image border were treated separately in the uniformity analysis, as it was reported earlier that border pixels may have a more severe depth dependence and consequently lower and broader photopeaks [23]. However, this effect is not evident in Figs. 2 and 3, where the ‘best’ border pixel (D) has a comparable response to the ‘best’ central pixel (A). The energy resolution of our detector is probably dominated by other factors than the pixel location, and the border non-uniformity is mainly determined by septum penetration and other effects.

Some degree of non-uniformity is present for all gamma camera systems, and the integral uniformity for quality control is usually calculated after uniformity correction has been applied. The integral uniformities obtained after correction in Table 3 are within the 5% acceptance limit recommended for conventional gamma camera systems [35].

The detector’s maximum count rate has been found to be approximately 71,000 cps, and no paralysable behaviour was found. The corresponding maximum activities of ^177^Lu were estimated to be between 700 MBq (MEGP) and 100 MBq (LEHS). These activities are only approximate since the detected count rate varies with the measurement geometry, the presence of attenuating material, and any activity located outside the FOV (Fig. 7).

As shown in Fig. 4, the spatial resolution differs between the collimators and varies slightly between the energy windows. The higher FWHM for the 208-keV energy window compared to the 113-keV window is consistent with analytical expressions for the spatial resolution [36], which explain the increase in FWHM with higher energies as a reduction in the effective collimator hole length due to less attenuation and an increasing penetration fraction. The poorer resolution of the 55-keV peak is probably related to the interference from photons that have scattered in the collimator, characteristic X-rays from the collimator, and tailing effects from the 113-keV and 208-keV photons.

Results for the hand-held camera in Fig. 4 refer to the spatial resolution in the central FOV. At the periphery of the FOV, the spatial resolution is expected to be similar, as the collimator is the major determining factor and all collimator holes have the same dimensions and are paired one-to-one with the anode elements. In preliminary measurements near the FOV border, the spatial resolution was found to be similar as those in the central FOV (data not shown), thus verifying this assumption.

In Fig. 4, the spatial resolution for a conventional gamma camera equipped with an MEGP collimator is plotted for comparison. At distances greater than 50 mm, the resolution of the conventional camera is better than that of the hand-held camera, while at short distances, the hand-held camera has a superior resolution. The poorer resolution at large source-collimator distances is probably related to the collimator hole lengths (Table 1), which are shorter than those of conventional gamma camera collimators. The better resolution at short distances is related to the image-formation process. The hand-held camera uses its small discrete anode elements to estimate photon interaction locations, which is expected to yield a better intrinsic resolution than the photomultiplier tube arrays of conventional cameras.

The system sensitivities obtained (Table 4) can be compared with those of a conventional camera [17]. Generally, with any given collimator, the hand-held camera has higher sensitivities than the conventional camera. This is probably due to the collimator design which results in a higher geometric acceptance [36]. The low-energy tailing also affects the sensitivity for the hand-held camera, which is decreased for 208 keV. For the lower energy windows, the sensitivity may be increased or decreased depending on whether the count contribution from the 208-keV tail is higher or lower than the loss of counts due to tailing of the particular energy.

Visually, the images from the hand-held camera in Fig. 9 appear to give a better detail resolution than the image from the conventional camera, and the apparently uniform distribution of [ ^177^Lu]Lu-DOTA-TATE is non-uniform in the images from the hand-held camera. This difference can be attributed to the very short source-collimator distance, which has been made possible by the small size of the hand-held gamma camera. At such short distances, the hand-held gamma camera has a favourable spatial resolution (Fig. 4). A limitation with the image-based comparison in Fig. 9 is that both the conventional and hand-held acquisitions were made in planar mode. The observed uptake patterns are thus also affected by the object extension and superposition of activity located at different depths. It would be of interest to perform detailed phantom measurements, with differently shaped objects, located at different depths to represent different amounts of attenuation and scatter, to further investigate the hand-held detector’s ability to identify non-uniform uptake patterns in practical applications and to perform activity quantification. However, such investigations were considered to be outside the scope of the current paper, in which we mainly focused on the detector characteristics. Another aspect of practical importance is insufficient detector shielding, which in our measurements was most apparent for the 208-keV energy window, and slightly also for 55 keV. For this reason, we have constructed a lead cover to be mounted on the detector, which is currently under evaluation.

## Conclusions

Based on our characterising measurements, we conclude that the hand-held camera works well for imaging of ^177^Lu in small, superficially located structures with modest activity in surrounding tissues and may in such situations be a more accessible alternative to conventional gamma cameras or small-animal imaging devices. The use of a 113-keV energy window in combination with an LEHR or MEGP collimator was found to provide the best results, with regard to the system spatial resolution, detector shielding, and septal penetration.

## Supplementary information

**Additional file 1** appendix.pdf. Contains *Appendix 1*, *Appendix 2* and *Appendix 3*, referenced in the main article text. *Appendix 1* presents more details on the energy resolution estimation procedure. *Appendix 2* presents peak drift values for all nuclides and photopeaks analysed. *Appendix 3* presents data on the noise levels in the flat-field images acquired for uniformity analysis.

## Data Availability

The camera characterisation measurements are available upon request to the corresponding author, Daniel Roth (daniel.roth@med.lu.se).

## Abbreviations

CZT: Cadmium zinc telluride
FOV: Field of view
LEHR: Low energy high resolution
LEHS: Low energy high sensitivity
MEGP: Medium energy general purpose
FWHM: Full width at half maximum
FWTM: Full width at tenth maximum
MCA: Multichannel analyser
ROI: Region of interest
TEW: Triple-energy window
