# Supplementary material for: Characterisation of a hand-held CZT-based gamma camera for 177Lu imaging
Source: EJNMMI Phys. 2020 Jul 8;7:46. doi: 10.1186/s40658-020-00313-y (PMC7343677; doi:10.1186/s40658-020-00313-y)
Supplement: Supplementary file 1 — Additional file 1 appendix.pdf. Contains Appendix 1, Appendix 2 and Appendix 3, referenced in the main article text. Appendix 1 presents more details on the energy resolution estimation procedure. Appendix 2 presents peak drift values for all nuclides and photopeaks analysed. Appendix 3 presents data on the noise levels in the flat-field images acquired for uniformity analysis. [file 40658_2020_313_MOESM1_ESM.pdf]

Appendix 1

The estimation of the energy resolution was made in a two-step process. Table S1 lists the energy intervals used for the first curve fit (Eq. 4). Energy intervals were chosen for each radionuclide based on the observed widths for the analysed peaks, with larger percentage intervals for the lower energies. The intervals were also adapted to exclude nearby peaks or other spectrum features that would otherwise interfere.

Table S1: Initial energy intervals, specified in values relative to the emission energy.

Nuclide	Interval
^{99m}Tc	−3 % to 10 %
^{111}In	−3 % to 10 %
^{131}I	−3 % to 5 %
^{177}Lu	−3 % to 15 %
^{22}Na	−3 % to 10 %
^{54}Mn	−3 % to 5 %
^{57}Co	−3 % to 8 %
^{133}Ba	−3 % to 20 %
^{137}Cs	−3 % to 5 %
^{241}Am	−6 % to 20 %

The initial estimates for a_0 and a_1 in Eq. 4 were obtained by smoothing the spectrum with a 10 keV wide mean-value filter and determining the value and position for the peak maximum. The initial estimate of a_2 was set to a value corresponding to a Gaussian FWHM of 6 keV. The initial values for a_3, a_4 and a_5 were zero.

A second curve fit (Eq. 5) was then performed for each peak separately within an energy interval to $[a_1 - p \cdot a_2, E_u]$. Initial estimates of a_1 and a_2 were obtained from the fit of Eq. 4, and the value of p was set to 1. The energy intervals obtained are presented in Table S2.

Table S2: Adjusted energy intervals used in the second curve fit (Eq. 5). Parameters a_1 and a_2 refer to the values obtained from the first curve fit (Eq. 4). Intervals are expressed in percentages relative to the emission energy.

Low-energy mode

Nuclide	Emission energy	a_1	a_2	Adjusted interval
^{99m}Tc	140.5	139.6	2.7	−2.6 % to 10.0 %
^{111}In	171.3	169.7	2.7	−2.5 % to 10.0 %
^{177}Lu	112.9	113.1	2.5	−2.0 % to 15.0 %
^{177}Lu	208.4	206.6	3.2	−2.4 % to 15.0 %
^{57}Co	122.1	122.0	2.5	−2.2 % to 8.0 %
^{57}Co	136.5	135.9	2.5	−2.3 % to 8.0 %
^{133}Ba	81.0	81.1	2.6	−3.0 % to 20.0 %
^{241}Am	59.5	60.2	2.5	−3.1 % to 20.0 %

High-energy mode

Nuclide	Emission energy	a_1	a_2	Adjusted interval
^{99m}Tc	140.5	140.6	3.0	−2.0 % to 10.0 %
^{111}In	171.3	170.5	3.2	−2.3 % to 10.0 %
^{131}I	284.3	282.9	3.5	−1.7 % to 5.0 %
^{131}I	364.5	362.4	4.0	−1.7 % to 5.0 %
^{177}Lu	112.9	113.5	3.0	−2.1 % to 15.0 %
^{177}Lu	208.4	207.0	3.5	−2.4 % to 15.0 %
^{22}Na	511.0	511.5	4.8	−0.8 % to 10.0 %
^{54}Mn	834.8	832.1	6.6	−1.1 % to 5.0 %
^{57}Co	122.1	121.9	3.0	−2.6 % to 8.0 %
^{57}Co	136.5	135.7	3.0	−2.8 % to 8.0 %
^{133}Ba	81.0	82.2	2.8	−1.9 % to 20.0 %
^{137}Cs	661.7	658.8	5.2	−1.2 % to 5.0 %
^{241}Am	59.5	60.7	3.1	−3.3 % to 20.0 %

Appendix 2

Table S3 summarises the observed photopeak drifts for all nuclides included in the temperature dependence analysis.

Table S3: Drifts in photopeak position during detector warm-up.

Nuclide	Photon energy [keV]	Peak drift [keV/°C]
^{177}Lu	∼55	0.17
^{241}Am	59.5	0.19
^{177}Lu	112.9	0.29
^{57}Co	122.1	0.31
^{57}Co	136.5	0.36
^{111}In	171.3	0.46
^{177}Lu	208.4	0.53

Appendix 3

Table S4 indicates the number of counts in all flat-field images used in the uniformity analysis. The data is presented as the average number of counts per pixel over the entire image. The relative standard deviation associated with this count level is presented as well, calculated as $\sqrt{n}/n$, where n is the number of counts.

Table S4: Average counts/pixel for all flat-field measurements. The relative standard deviation associated with this count level is given in parentheses.

		55 keV	113 keV	208 keV
	MEGP	76480 (0.36 %)	102541 (0.31 %)	74651 (0.37 %)
Reference	LEHR	52798 (0.44 %)	69797 (0.38 %)	89085 (0.34 %)
measurements[2]	LEHS	180203 (0.24 %)	236450 (0.21 %)	274884 (0.19 %)
	OPEN[1]	129694 (0.28 %)	182069 (0.23 %)	80734 (0.35 %)
	MEGP	15597 (0.80 %)	21027 (0.69 %)	15264 (0.81 %)
Control	LEHR	18608 (0.73 %)	24365 (0.64 %)	30699 (0.57 %)
measurements[3]	LEHS	86191 (0.34 %)	114338 (0.30 %)	134406 (0.27 %)
	OPEN[1]	164226 (0.25 %)	230424 (0.21 %)	102275 (0.31 %)

[1]Open field cover

[2]Measurements used to derive uniformity-correction matrices

[3]Measurements used to calculate intrinsic uniformity with uniformity-correction
